# Acquisition and maintenance of motor memory through specific motor practice over the long term as revealed by stretch reflex responses in older ballet dancers

**DOI:** 10.14814/phy2.14335

**Published:** 2020-01-20

**Authors:** GeeHee Kim, Tetsuya Ogawa, Hirofumi Sekiguchi, Kimitaka Nakazawa

**Affiliations:** ^1^ Graduate School of Arts and Sciences The University of Tokyo Komaba Tokyo Japan; ^2^ Research Institute National Rehabilitation Center for Persons with Disabilities Namiki Tokorozawa Japan; ^3^ Sports Management Program Faculty of Business and Information Sciences Jobu University Isesaki Gunma Japan

**Keywords:** aging, ballet practice, motor memory, motor training, stretch reflex

## Abstract

The present study addressed whether motor memory acquired earlier in life through specific training can be maintained through later life with further training. To this end, the present study focused on the training effect of a specific ballet practice and investigated the spinally mediated stretch reflex responses of the soleus muscle in ballet dancers of upper‐middle to old age (60.6 ± 5.4 years old) with experience levels of 28.4 ± 7.4 years (“older ballet” group). Comparisons were conducted with a group of young ballet dancers (“young ballet” group) and groups of both young and older individuals without weekly participation in physical activities (“young sedentary” and “older sedentary” groups). The results revealed natural age‐dependent changes, with reflex responses being larger in older sedentary than in young sedentary individuals. A training‐induced effect was also observed, with responses being smaller in ballet dancers than in sedentary groups of the same age. Furthermore, the responses were surprisingly smaller in the older ballet dancers than in the young sedentary group, at an equivalent level to that of the young ballet dancers. The influence of training, therefore, overcame the natural age‐dependent changes. On the other hand, the onset latencies of the responses showed a solely age‐dependent trend. Taken together, the present is the first to demonstrate that the motor memories in the spinal cord acquired through specific ballet training earlier in life can be maintained and carried forward in later life through further weekly participation in the same training.

## Graphical abstract

With the knowledge that human motor system undergoes functional changes after participation in specific motor training, the present study first demonstrated that the motor memories in the spinal cord acquired through participation in specific ballet training earlier in life can be maintained and carried forward in later life through further weekly participation in the training.

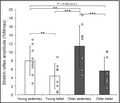

## 1 INTRODUCTION

It is well accepted that the human motor system undergoes functional changes associated with age. In the reflex system, specifically, the ability to modulate the spinal reflex via presynaptic inhibition has been proposed to be attenuated in elderly subjects (Baudary, Maerz, & Enoka, 2009; Butchart, Farquhar, Part, & Roberts, 1993; Earles, Vardaxis, & Koceja, 2001; Koceja & Mynark, 2000). For instance, the degree of modulation in the electrically elicited H‐reflex was smaller in elderly than in young individuals when the H‐reflex was conditioned by changing the posture from supine to upright (Koceja & Mynark, 2000) or by applying a preceding conditioning stimulus using electricity (Baudary et al., 2009; Earles et al., 2001).

On the other hand, use‐dependent plasticity of the central nervous system, particularly in the motor system, has been demonstrated extensively both in animals and in humans over the past few decades. In animal studies, the best example of this use‐dependent plasticity is probably the modulation of the spinal monosynaptic reflex, which is induced by operant conditioning (Wolpaw, O'Keefe, Noonan, & Sanders, 1986; Chen & Wolpaw, 1995; Wolpaw, 1987; Carp, Tennissen, Chen, & Wolpaw, 2006). Throughout reward‐based training over weeks and months, the amplitude of the spinal stretch reflex or H‐reflex can be either up‐regulated or down‐regulated persistently in monkeys (Wolpaw, 1987; Wolpaw et al., 1986), rats (Chen & Wolpaw, 1995), and mice (Carp et al. 2006).

In humans, along with a few longitudinal studies including those using operant conditioning (Thompson, Chen, & Wolpaw, 2009; Thompson, Pomerantz, & Wolpaw, 2013), most studies on use‐dependent plasticity involved cross‐sectional comparisons of different subject groups with different physical backgrounds. In these comparisons, the magnitudes of spinally mediated reflex responses were characterized by the types of physical training the subjects participated in (Rochcongar, Dassonville, & Le Bars, 1979; Casabona, Polizzi, & Perciavalle, 1990; Maffiuletti et al., 2001; Ogawa et al., 2009). Among the various types of physical training, the present study focuses on ballet dancing. For maintenance of their posture during ballet dancing, dancers have to co‐contract antagonistic muscles, which in turn facilitates the presynaptic inhibition of the Ia afferent terminal (Nielsen & Kagamihara, 1993). The amplitude of spinal reflex responses has thus been demonstrated to be much smaller in ballet dancers than in the respective other subject groups that were tested, both for the electrically induced H‐reflex (Nielsen, Crone, & Hultborn, 1993) and for the Achilles tendon tapping reflex (T‐reflex) (Goode & Van Hoven, 1982).

Considering these activity‐dependent reductions in the amplitude of reflex responses as a possible consequence of enhanced presynaptic inhibition, the present study aimed to determine whether the acquired motor memory could be maintained with further weekly participation in this specific training throughout life. The present study then conducted cross‐sectional comparisons of the spinal reflex responses among young and old individuals either with or without long‐term weekly motor skill training in ballet dancing. Differences in the reflex responses associated with age and motor skill training history were discussed in terms of acquisition and long‐term maintenance of motor memory in spinal pathways.

## 2 METHODS

### 2.1 Subjects

A total of 36 volunteers were studied in four groups: nine young ballet dancers (all female, age; mean ± *SD*: 21.1 ± 2.8 years), nine young sedentary individuals (all female, 23.1 ± 1.8 years), nine older ballet dancers (all female, 60.5 ± 5.3 years), and nine older sedentary individuals (one female and eight male, 69.4 ± 6.7 years old). Table 1 summarizes the physical characteristics of the subjects. In both sedentary groups (young and older), the subjects did not participate in physical activities beyond those associated with typical daily living. In the group of ballet dancers, the time elapsed since the first participation in ballet practice was 15.6 ± 3.2 years in the young group and 28.4 ± 7.4 years in the older group. In addition, the subjects in these ballet groups participated in ballet practice (average of 4.5 ± 1.1 (*SD*) hours per week in the young group and 5.0 ± 2.6 hr per week in the older group) at the time of measurement. None of the subjects had any known history of neurological or orthopedic disorder. All subjects gave their written informed consent to undergo the experimental procedure. Experimental procedures were approved by the local ethics committee of the National Rehabilitation Center for Persons with Disabilities, Japan and were conducted in accordance with the Declaration of Helsinki.

**Table 1:** Physical characteristics of the subjects in each subject group

	Subjects	Gender	Age (years old)	Height (cm)	Weight (kg)	Time since first participation (years)	Ballet practice (hours/week)
Young sedentary	1	F	24	158	40	–	–
	2	F	25	160	54	–	–
	3	F	26	161	51	–	–
	4	F	22	166	56	–	–
	5	F	23	163	55	–	–
	6	F	24	156	48	–	–
	7	F	21	156	48	–	–
	8	F	22	154	49	–	–
	9	F	21	166	56	–	–
	Mean ± *SD*		23.1 ± 1.8	160 ± 4.4	50.8 ± 5.2	–	–
Young ballet	1	F	20	164	55	16	4.0
	2	F	25	163	50	17	5.0
	3	F	25	165	46	18	5.0
	4	F	19	161	48	13	4.5
	5	F	18	162	50	8	6.0
	6	F	19	149	40	16	4.5
	7	F	24	160	53	18	5.0
	8	F	21	165	51	18	2.0
	9	F	19	153	54	16	4.5
	Mean ± *SD*		21.1 ± 2.8	160.2 ± 5.6	49.7 ± 4.6	15.6 ± 3.2	4.5 ± 1.1
Old sedentary	1	M	66	166	60	–	–
	2	M	69	165	64	–	–
	3	M	68	155	58	–	–
	4	M	65	162	60	–	–
	5	M	65	170	68	–	–
	6	M	64	165	58	–	–
	7	F	85	148	45	–	–
	8	M	68	162	63	–	–
	9	M	75	168	67	–	–
	Mean ± *SD*		69.4 ± 6.7	162.3 ± 6.9	60.3 ± 6.8	–	–
Old ballet	1	F	63	157	48	28	4.5
	2	F	58	156	43	28	4.0
	3	F	65	157	45	25	3.0
	4	F	67	160	51	28	3.0
	5	F	67	148	46	33	10.0
	6	F	53	153	44	15	3.0
	7	F	60	160	51	42	9.0
	8	F	59	162	45	24	4.5
	9	F	53	163	53	33	4.0
	Mean ± *SD*		60.6 ± 5.4	157.3 ± 4.7	47.3 ± 3.6	28.4 ± 7.4	5.0 ± 2.6

### 2.2 Experiments

In the experiment, stretch reflex responses evoked in the plantarflexor soleus muscle were recorded. A specially designed device was used to induce the responses (Senoh Corp.) (Kawashima et al., 2004; Nakazawa, Kawashima, & Akai, 2006; Ogawa et al., 2009). Each subject sat comfortably in the chair of the device with the hip, knee, and ankle joint angles fixed at 100°, 120°, and 90° (bilaterally), respectively, and with both feet placed on the footplate. Only the right foot was tightly fixed to the footplate connected to a servo‐controlled torque motor. The axis of rotation of the foot plate was adjusted to the center of the ankle joint. With the subjects completely at rest, the stretch reflex responses in the soleus muscle were elicited by sudden and quick toe‐up rotation of the footplate with displacement of 10°. Four different rotational stimuli with settings of 100, 200, 300, and 400 deg/s, respectively were used. For each trial, one of the four rotational stimuli was presented in a random order for a total of 48 trials (12 trials each). The intervals between each trial were pseudorandom and ranged between 5 and 9 s.

### 2.3 Recordings and data analysis

Electromyographic (EMG) responses were recorded in the soleus and tibialis anterior muscles of the right leg using bipolar Ag/AgCl surface electrodes (10‐mm diameter, 15‐mm center‐to‐center inter‐electrode distance). The raw EMG signals were band‐pass filtered (15–3 kHz) and amplified using a conventional bioamplifier (AB‐621B; Nihon Kohden Corp.). The EMG responses, along with the angle and torque signals of the footplate, were digitized at a sampling rate of 10 kHz, and were stored in a computer for later off‐line analysis.

The onset of the stretch reflex response was defined as the moment when the EMG activity level reached a level higher than the mean baseline level (50‐ms time window before the stimulus onset) plus three times its standard deviation in a full‐wave rectified waveform (Nakazawa et al., 2006). The stretch EMG responses of the soleus muscle (which is likely the M1 (spinal) component of the stretch reflexes) were calculated as the peak‐to‐peak amplitude (in a non‐rectified waveform) for 20 ms after the onset of the responses. The amplitudes of the stretch reflex responses are presented as values relative to the maximal motor response (Mmax) obtained by applying an electrical stimulus to the posterior tibial nerve.

For statistical analysis, two‐way analysis of variance (ANOVA) with repeated measures (Bonferroni's) was used for the stretch reflex amplitude and latency with factors of stretch velocity and subject groups. For comparisons of the stretch reflex amplitude and latency regardless of stretch velocity (that is, the mean of all 48 responses), one‐way ANOVA with repeated measures (Bonferroni's) was used. All data are presented as mean values and standard deviations (mean ± *SD*). The criterion for inferring statistical significance was *p* < .05.

## 3 RESULTS

**Figure 1:**
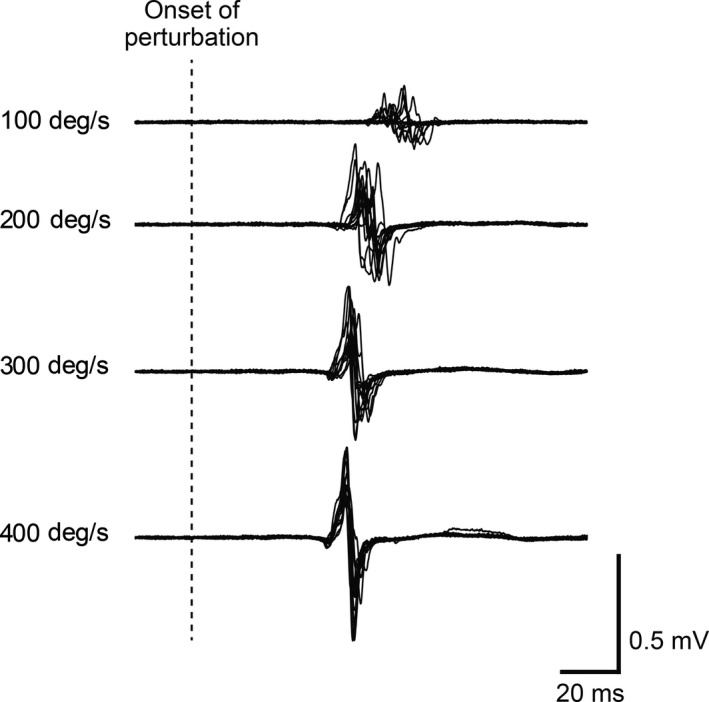
Representative EMG responses for the stretch reflex in a single subject from the older ballet group. Each set of raw EMG waveforms was obtained by superimposition of twelve stretch reflex responses evoked under each stretch velocity setting (100, 200, 300, and 400 deg/s). The vertical dotted line represents the onset timing of the footplate movement to evoke the responses

**Figure 2:**
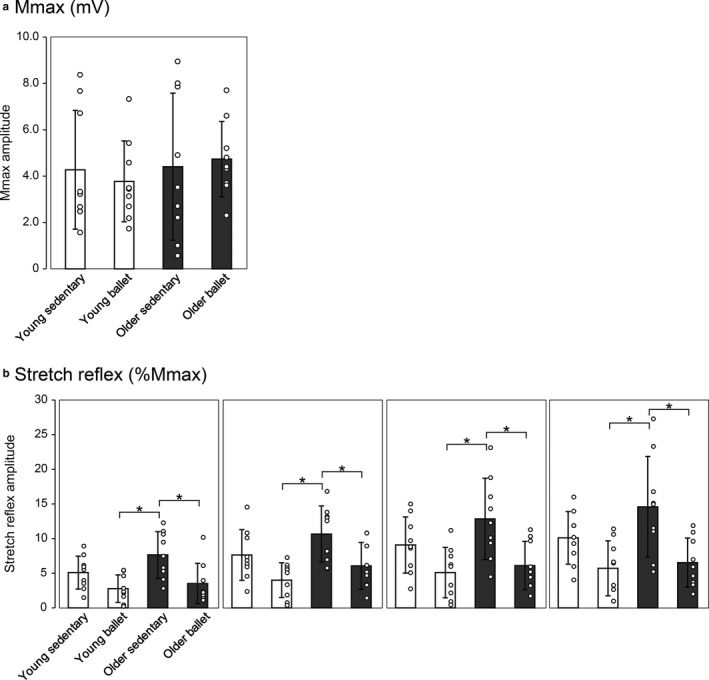
Comparisons of the Mmax (a) and stretch reflex responses (b) among the subject groups. In b, each panel represents the mean amplitude of stretch reflex responses in % of Mmax obtained under a specific velocity setting (100, 200, 300, and 400 deg/s). The scatter plots visualize the data for each subject. Error bars represent standard deviations (*SD*). Significant differences *p < .05, **p < .01

Figure 1 illustrates a typical example of the stretch reflex EMG responses of a single subject from the older ballet group. With the amplitude and the latency of the EMG responses depending on the angular velocity of the footplate, there are typically single twitch‐like bursts. On the basis of the calculation of the peak‐to‐peak amplitude of the responses, Figure 2 shows the comparisons of the Mmax (A) and the mean stretch reflex amplitudes in % of Mmax (B) among the four subject groups. There were no statistically significant differences in Mmax among the subject groups (*F* = 0.26, *df* = 3, *p* = .856). In Figure 2b, the panels show the results for the different stretch velocity settings. Generally, the stretch reflex responses tended to be larger in the older groups than in the young groups, and were smaller in the groups of ballet dancers than in the sedentary groups.

**Figure 3:**
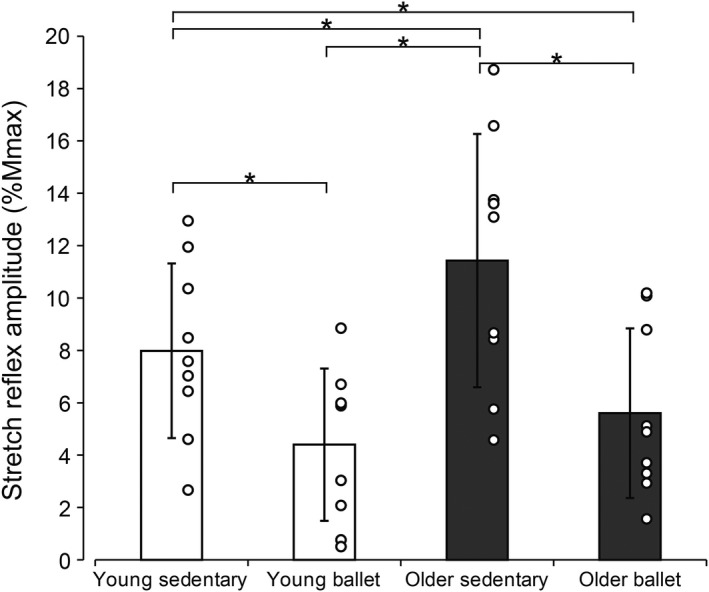
Mean values of the stretch reflex amplitudes obtained for all stretch velocities tested (including data from all velocities). The scatter plots visualize the data for each subject. Error bars represent standard deviations (*SD*). Significant differences **p < .01, ***p < .001. n.s. = not significant

Regarding the stretch reflex responses (Figure 2b), there was a significant main effect both for the subject group (*F* = 6.549, *df* = 3, *p* = .001) and the stretch velocity (*F* = 43.778, *df* = 3, *p* < .001) and an interaction between the two (*F* = 2.251, *df* = 9, *p* = .025). Post‐hoc comparisons revealed that the mean amplitude in both dancer groups (young and older) was always smaller than that in the older sedentary groups, for all stretch velocities (*p* = .004 (100 deg/s), *p* = .002 (200 deg/s), *p* = .004 (300 deg/s), and *p* = .003 (400 deg/s) for young ballet versus older sedentary; *p* = .017 (100 deg/s), *p* = .049 (200 deg/s), *p* = .015 (300 deg/s), and *p* = .009 (400 deg/s) for older ballet vs. older sedentary). Further differences in the stretch reflex amplitude among the groups are presented in Figure 3, in which the mean reflex amplitude across different stretch velocities was calculated for each group. The one‐way ANOVA revealed a significant main effect (*F* = 19.794, *df* = 3, *p* < .001). In the post‐hoc assessment, the mean amplitude was generally larger in the older group than in the young group in the comparison between the two sedentary groups (*p* = .004 for young sedentary vs. older sedentary), while only a tendency of the same pattern (without statistical significance) was observed in the comparison between the young sedentary group and older ballet dancers (*p* = .092). The response in the older sedentary group was also larger than that in the young ballet group (*p* < .001). The mean amplitude for the ballet dancers was always smaller than that in the age‐matched sedentary groups (*p* = .002 for young sedentary vs. young ballet, and; *p* < .001 for older ballet vs. older sedentary).

**Figure 4:**
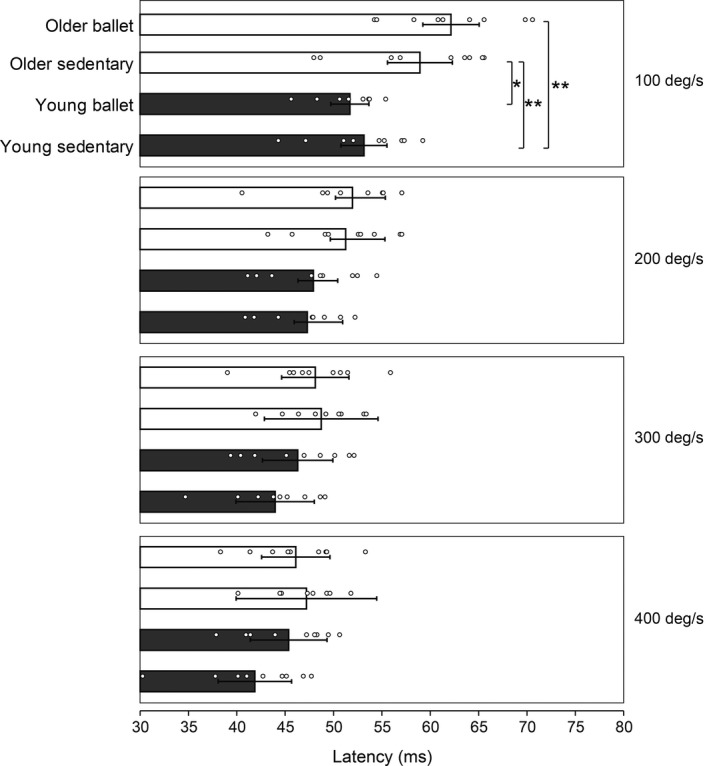
Comparisons of the mean latency of the stretch reflex responses in each subject group in accordance with the stretch velocity settings. The scatter plots visualize the data for each subject. Error bars represent standard deviations (*SD*). Significant differences *p < .05, **p < .01

**Figure 5:**
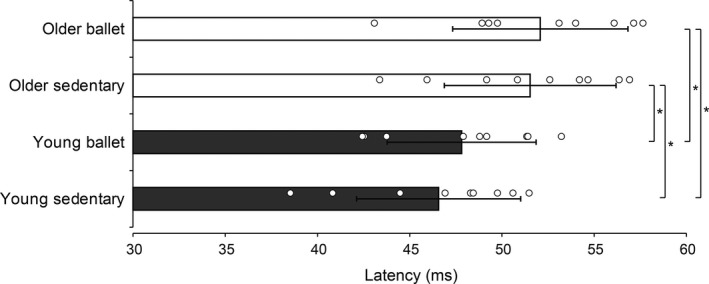
Intergroup comparisons of the mean stretch reflex latencies regardless of stretch velocity (including data from all velocities). The scatter plots visualize the data for each subject. Error bars represent standard deviations (*SD*). Significant differences *p < .05, **p < .01. n.s. = not significant

Figure 4 shows the latency of the stretch reflex responses. Illustrated in the typical EMG waveform, the latency was generally reduced as the stretch velocities increased (see Figure 1). The older groups generally showed longer latencies than the young groups. Two‐way ANOVA revealed a significant main effect both for the subject group (*F* = 3.316, *df* = 3, *p* = .032) and the stretch velocity (*F* = 228.741, *df* = 3, *p* < .001) and a significant interaction (*F* = 7.453, *df* = 9, *p* < .001). Post‐hoc analysis revealed further differences in variables between groups, particularly for the stretch velocity of 100 deg/s (*p* = .002 for young sedentary vs. older ballet, *p* = .047 for young ballet vs. older sedentary, and *p* = .008 for young sedentary vs. older sedentary). Regardless of stretch velocity (Figure 5), there was a significant main effect (*F* = 6.296, *df* = 3, *p* < .001) of subject group. In the post‐hoc analyses, the mean latency in the older ballet group was significantly larger than that in both young groups (*p* = .003 vs. young sedentary and *p* = .037 vs. young ballet), respectively. Similarly, the mean latency was significantly larger in the older than in the young sedentary group (*p* = .009), while there was only a tendency of a difference (*p* = .100) between the older sedentary and the young ballet groups.

## 4 DISCUSSION

To investigate the nature of the central nervous system (CNS) in terms of both the acquisition and the maintenance of motor memory in the spinal cord through specific motor training, spinally mediated stretch reflex responses were compared among young and older subjects either with or without long‐term weekly participation in ballet practice. Our results show a significant age‐dependency in that the amplitude of the stretch reflex response was significantly larger in the older group than in the young group in a comparison between subject groups that did not participate in ballet practice (young sedentary vs. older sedentary). The results were also dependent on participation in ballet practice, with responses being smaller in subjects who participated in ballet practice than in those who did not when comparisons were made between subject groups of similar ages (young sedentary vs. young ballet, older sedentary vs. older ballet). *F*urthermore, the responses in the subject groups who participated in ballet training were smaller than those in both sedentary groups, not only for the young ballet group (young ballet vs. young sedentary, young ballet vs. older sedentary) but also for the older ballet group (older ballet vs. young sedentary, older ballet vs. older sedentary). The results for the onset latency of stretch reflex responses only showed an age‐dependent trend. These results therefore suggest that motor memories in the spinal cord acquired through participation in specific ballet training earlier in life can be maintained and carried forward in life through further weekly participation in similar training.

### 4.1 Methodological considerations

Regarding the neural changes associated with aging, particularly those involving the spinally mediated reflex responses, previous studies have reported controversial results in terms of the reflex amplitude to given stimuli. For example, the magnitude of the stretch reflex has been reported to be reduced with age (Corden & Lippold, 1996; Lin & Sabbahi, 1998) in some studies while it remained unchanged in others (DeVries, Wiswell, Romero, & Heckathorne, 1985; Kawashima et al., 2004; Nardone, Siliotto, Grasso, & Schieppati, 1995). This controversy, however, seems largely dependent on the experimental conditions such as differences in the muscles tested (such as hand, wrist, or foot), state of the muscles (during rest or while standing), and modality of the stimuli (tapping the tendon or passive joint movement). It is therefore important to note that the present results cannot be directly compared with those reported in previous studies. It should be noted, therefore, that the intention of the present study is not to provide a definitive answer to the controversy in the literature. The present study aimed to address the capability of the human spinal cord to acquire and maintain motor memories based on comparisons of stretch reflex responses obtained in these particular experimental settings among subject groups with different physical backgrounds.

Another factor underlying the controversy and making intergroup comparisons difficult is related to the methodological issues associated with the analysis of the obtained responses. While most studies conducted intergroup comparisons of stretch reflex responses on the basis of responses obtained using particular stimuli (Corden & Lippold, 1996; Nardone et al., 1995), the magnitude of the obtained responses could be largely dependent on the input (i.e., pedal or ankle rotation speeds, hammer height of tendon tapping, magnitude of electric stimulus)–output (response) relationship of the given pathway. Therefore, similar results cannot be guaranteed for different stimuli if the input–output relationship differs between the individuals or groups under comparisons (Kawashima et al., 2004; Lin & Sabbahi, 1998; Ogawa et al., 2009). The present study, therefore, utilized several different stimuli (rotational velocities of the foot plate to stretch the soleus muscle) to comprehensively elucidate the potential differences among each subject and the subject group.

Regarding the gender of each subject group, the older sedentary group included a majority of male subjects, while all other groups consisted of only female subjects. We do however not assume that the inclusion of the male subjects in the older sedentary group had a major influence on the overall results of the current study, due to the facts that one female subject in the older sedentary group showed a higher stretch reflex amplitude than the other eight male subjects, which is in line with results of a previous study where females showed higher Hmax/Mmax ratios than male subjects (Hoffman, Norcross, & Johnson, 2018).

### 4.2 Neural changes associated with age

Consequently, the amplitude of the stretch reflex response was prominently larger in the older in comparison to the young group when the sedentary groups were compared. This larger response in the older group can be partially attributed to the attenuation of inhibition of the presynaptic terminal of the monosynaptic reflex pathway (Baudary et al., 2009; Butchart et al., 1993; Earles et al., 2001; Koceja & Mynark, 2000). This is also in accordance with the exaggerated stretch reflex responses noted in spastic patients, which could result from the effect of decreased presynaptic inhibition on the Ia afferent terminal (Aymard et al., 2000; Lamy et al., 2009). Another possible reason underlying the greater stretch reflex response in the older individuals stems from the mechanical features of the musculoarticular structure in the stretched muscle. With less maximal elongation and strain of the tendon structure in elderly individuals (Kubo et al. 2007), an earlier study demonstrated parallel increases in stretch reflex responses and musculoarticular stiffness in prepubescent children (Grosset, Mora, Lambertz, & Perot, 2007). These possible changes in the mechanical properties of the musculoarticular structures seem to be in conflict with the results in the latency of the stretch reflex responses, as the older group (with stiffer musculoarticular structure) showed generally greater latencies (Figure 4). The stretch reflex latency is, however, not simply dependent on the mechanical properties of the musculoarticular structure but can also be affected by the physiological changes in the spinal circuits. Indeed, the latency of the H‐reflex response was shown to be delayed in older than in younger individuals, although this electrically induced reflex response does not include the mechanically induced stretch of the target muscle (Scaglioni, Narici, Maffiuletti, Pensini, Martin, 2003). The delayed reflex latencies in the older subjects in the present study could be related to morphological changes in nerve fibers, such as a decline in myelinated fiber density (Jacobs and Love, 1985), which in turn results in decreased nerve conduction velocity (Verdu, Ceballos, Viches, & Navarro, 2000). In addition to the conduction velocity of the neural fibers, the length of the pathway could play a significant role in the onset latency of the stretch reflexes; however, there were no major differences in subject height which indicates the pathway length among the subject groups (Table 1).

### 4.3 Influence of motor training on neural plasticity

The possibility of plastic changes in the human motor system with respect to physical training has been addressed extensively in the literature. On the basis of cross‐sectional comparisons among subject groups with different histories of physical training, responses of the soleus H‐reflex were generally larger in aerobically trained athletes and were, in contrast, smaller in those who received anerobic‐type physical training (Rochcongar et al. 1979; Casabona et al., 1990; Maffiuletti et al., 2001). This aspect of plastic changes is sometimes obvious as differences in the modulation patterns of the spinally mediated stretch reflex among highly trained endurance runners upon different activation levels of the stretched muscle (Ogawa, Kawashima, Suzuki, & Nakazawa, 2012a). Moreover, the stretch reflexes in lower leg muscles were different between the legs within individuals after routine training in stereotypical physical training that emphasizes differing uses of two legs (Ogawa, Kawashima, Suzuki, & Nakazawa, 2012b).

Among the various types of physical training, repetitive participation in ballet dancing has particularly been found to reduce the responses of the spinally mediated reflex responses, as demonstrated in the smaller responses of the electrically induced H‐reflex (Nielsen et al., 1993) and Achilles tendon tapping reflex (T‐reflex) (Goode & Van Hoven, 1982). It was postulated that ballet dancers perform co‐contraction of antagonistic muscles to maintain balance during classical ballet postures (Nielsen & Kagamihara, 1993). The reduction in the reflex responses results from an increase in presynaptic inhibition during such co‐contraction (Nielsen & Kagamihara, 1993). In the present results as well, it is possible that an increase in presynaptic inhibition and therefore a reduction in the synaptic transmission at the Ia afferent terminal due to weekly participation in the specific training resulted in the smaller reflex responses in the groups of ballet dancers. However, since the measurement in the present study was conducted while the subjects were at rest where presynaptic inhibition is low, the influence of presynaptic inhibition can be considered insignificant. In addition, regarding the mechanically induced stretch reflex, it is known that its amplitude is less susceptible to presynaptic inhibition in comparison to the electrically evoked H‐reflex (Morita, Petersen, Christensen, Sinkjaer, & Nielsen, 1998). However, the present results show that the amplitude of the stretch reflex responses were significantly smaller in the groups of ballet dancers (both young and older) than in the sedentary groups, and there were no differences between the groups of ballet dancers (Figure 3). Although the capability of the motor system to reduce stretch reflex responses, particularly through presynaptic inhibition, has been demonstrated to be attenuated in the elderly (Baudary et al., 2009; Butchart et al., 1993; Earles et al., 2001; Koceja & Mynark, 2000), the present results suggested that specific motor memories acquired earlier in life can be maintained and carried forward later through weekly participation in the same specific training.

### 4.4 Conclusion

The present results based on intergroup cross‐sectional comparisons of the amplitude of stretch reflex responses demonstrated the capability of the human motor system both in terms of the acquisition and maintenance of motor memory through weekly participation in long‐term specific training. In contrast to the functional aspect, only age‐dependent differences were observed in the onset latencies of the stretch reflex responses, possibly a reflection of the physiological aspect of the neural pathway. Considering our knowledge of the attenuation of motor function in the elderly, the present findings postulate a significant role of weekly participation in motor training throughout life in the maintenance of adequate motor function and therefore the maintenance of health and quality of life in older populations.

## CONFLICTS OF INTEREST

The authors declare that no competing interests exist.
